# Prevalence and distribution of soil-transmitted helminth infection in free-roaming dogs in Bali Province, Indonesia

**DOI:** 10.14202/vetworld.2021.446-451

**Published:** 2021-02-22

**Authors:** Kadek Karang Agustina, Made Suma Anthara, Nengah Anom Adi Nugraha Sibang, Wayan Adi Rinta Wiguna, Jendra Krisna Apramada, Wayan Nico Fajar Gunawan, Ida Bagus Made Oka, Made Subrata, Nengah Kerta Besung

**Affiliations:** 1 Department of Public Health, The Faculty of Veterinary Medicine, Udayana University, Denpasar, Bali 80225, Indonesia; 2 Department of Veterinary Clinic, The Faculty of Veterinary Medicine, Udayana University, Denpasar, Bali 80225, Indonesia; 3 Department of Parasitology, The Faculty of Veterinary Medicine, Udayana University, Denpasar, Bali 80225, Indonesia; 4 Department of Epidemiology, The Public Health Study Program, Faculty of Medicine Udayana University, Denpasar, Bali 80225, Indonesia; 5 Department of Microbiology, The Faculty of Veterinary Medicine, Udayana University, Denpasar, Bali 80225, Indonesia

**Keywords:** *Ancylostoma* spp, *Ascaris* spp, Bali, distribution, free-roam dogs, prevalence, soil-transmitted helminth, *Trichuris* spp

## Abstract

**Background and Aim:**

Several free-roaming dogs can be easily found in the public areas of Bali. They go out in search of foods and friends and defecate everywhere. In general, these groups of dogs do not receive good healthcare from their owners and are generally threatened by some disease-causing organisms, especially helminths. This study was conducted to identify and measure the prevalence of soil-transmitted helminths (STHs) that cause infection in free-roaming dogs in Bali Province, Indonesia.

**Materials and Methods:**

A total of 1611 fresh dog fecal samples were collected from all areas of Bali Province and subjected to qualitative fecal examination using flotation techniques to obtain STH eggs.

**Results:**

The incidence of STHs in free-roaming dogs was 38.36%. However, three types of STHs were identified, including *Ancylostoma* spp., *Ascaris* spp., and *Trichuris* spp. *Ancylostoma* spp. had the highest prevalence of 37.8%, followed by *Ascaris* spp. and *Trichuris* spp. at 6.02% and 0.87%, respectively. Multiple infections of these worms were also recorded. The polyparasitism prevalence of *Ancylostoma* spp. and *Ascaris* spp. was 3.85%, followed by that of *Ancylostoma* spp. and *Trichuris* spp. at 0.5% and that of *Ascaris* spp. and *Trichuris* spp. at 0.06%. There were no altitude-wise differences in the prevalence of STH infection.

**Conclusion:**

The prevalence of STHs was high in free-roaming dogs. This finding necessitates more serious attention as it affects both animal and public health.

## Introduction

Dogs cannot be separated from human life, and they are often referred to as the Best Friend of Man. Petting a dog, apart from being a hobby, can provide several benefits for the owner. According to some research, a dog used as a pet can have a positive impact on psychology and health [1]. In Bali, dogs are used as both pets and protectors of homes. Therefore, they are free to roam as they are terrestrial animals and the guardians of their territories [2]. The population of free-roaming dogs in Bali is quite high, with the prevalence reaching 69.71% [3,4] and 19.4% in rural and urban areas, respectively [2].

In contrast to the benefits provided, the maintenance patterns of allowing them to roam tend to have adverse effects on the health of the community as well as other animals [5]. As a result, several cases have been reported in the Bali community [6]. Dangerous dog-borne infections can be caused by viruses, bacteria, or parasites [7]. However, specific diseases caused by parasites do not attract the attention of the community, which is because they rarely cause clinical symptoms [8].

Moreover, parasitic worms that commonly infect dogs belong to the nematode species [9]. These types of worms include *Ancylostoma* spp., *Ascaris* spp., and *Trichuris* spp. [10]. They are commonly classified into soil-transmitted helminth (STH) groups [11]. Some types of STH worms that infect dogs have been reported as zoonotic agents, namely, *Ancylostoma ceylanicum*, *Ancylostoma caninum*, and *Toxocara canis* [12]. STHs infect both animals and humans through the intake of infective eggs or larvae. Therefore, dogs are known to play a crucial role in the transmission of parasites to other animals and humans [13].

In Bali, several studies have reported the prevalence of worms that infect dogs. For instance, *T. canis* was reported to infect Kintamani dogs in the Sukawana Village area with a prevalence of 22.22% [14]. The prevalence of *Ancylostoma* spp. in tourist areas was reported as 34% [15]. However, there are no reports on prevalence covering all areas in Bali. Therefore, this study was conducted to investigate the prevalence and type of STH worms in dogs that freely roam throughout the province of Bali, Indonesia.

## Materials and Methods

### Ethical approval

The study only used the dog fecal samples which were collected from the residential alley, street, or other public places. Hence, ethical approval was not necessary.

### Study period and location

This research was conducted from February to November 2019 in the province of Bali, Republic of Indonesia.

### Samples

An observational design with a cross-sectional method was used in this study, and the subjects were the free-roaming dogs in the province of Bali. The study sample was fresh dog feces collected from the subjects. Fresh stools were collected in the morning around the residential alley, street, or other public places and were stored in 10% formalin before being examined at the laboratory [16]. The Bali government has reported that the dog population in Bali was 647.386 in 2019. This number was the highest in Indonesia compared with other provinces. The majority of dogs are free-roaming (approximately 90%), and only 10% of the dog population is well managed [17]. The study sample was calculated using a formula described elsewhere [18], and a minimum number of 73 samples from each regency was required. A total of 1611 samples were collected from all areas of Bali Province and grouped according to regency into Badung, Gianyar, Klungkung, Bangli, Karangasem, Tabanan, Buleleng, and Denpasar city. In addition, they were categorized based on location elevation. Lowland and highland were determined by the altitude. More than 600 m above the sea level is categorized as highland, and less than 300 m is categorized as lowland [19].

### Stool examination

Stool examination was performed by a floating concentration method using a saturated NaCl substance [20]. The concentration McMaster techniques were used to measure the intensity of infection of each identified worm [21].

### Statistical analysis

The following formula was used to measure the prevalence of STH [22], and the data obtained are presented descriptively. Furthermore, the Chi-square test was used in comparing the prevalence of STH according to the landscape [23].


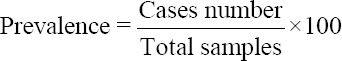


## Results and Discussion

This study demonstrated that the prevalence of STH infection in free-roaming dogs was high (38.36%) (Table-1). To the best of our knowledge, this is the first report on free-roaming dogs in Bali, Indonesia, and the obtained data correlated with the report on rural dogs living in farms around the Atlantic forest fragments in Brazil [23]. However, the prevalence determined in this study was lower than previous prevalence rates of 98.8% in Tacuarembo, Uruguay [24], 95% in Bangladesh [25], 93.1% in Durban and Coast, South Africa [26], 78.57% in Ilam Province of Iran [27], 61.8% in Italy [28], and 51% in Debre Zeit, Ethiopia [9]. In contrast, this prevalence was higher than previous prevalence rates of 21.5% in Mexicali County in Northwest Mexico [29], 16.5% in Calgary, Alberta [30], and 9.4% in Northern Germany [31]. Our study shows that free-roaming dogs in Bali spread and contaminate the environment by STH eggs. Therefore, this problem requires serious public and animal health attention because STHs can cause severe health problems in dogs [32], including retarded growth, reduced immune response to infectious diseases, and generalized ill health [33].

**Table-1:** The prevalence of STH infection in free-roam dogs and its distribution in Bali Province.

Regency	Total sample	STH	%
Bangli	192	97	50.52
Tabanan	290	129	44.48
Denpasar	168	74	44.05
Gianyar	199	84	42.21
Negara	136	57	41.91
Karangasem	175	63	36.00
Badung	139	41	29.50
Klungkung	163	43	26.38
Buleleng	149	30	20.13
Total	1,611	618	38.36

STH=Soil transmitted helminths

Hookworm infection was found to be the most frequent parasite in the investigated dog population, with a prevalence of 37.8%, followed by infections with *Ascaris* spp. and *Trichuris* whose respective prevalence rates were 6.02% and 0.87% (Tables 2 and 3). *Ancylostoma* spp. is known to be predominant in the small intestines of animals and humans, among which *A. ceylanicum*, *Ancylostoma braziliense*, and *A. caninum* are known as the disease-causing agents [32,33]. Their eggs, which are exclusively thrown, can inevitably infect the soil on which dogs defecate. Humans become infected when their skin comes in contact with the larvae and make penetration. This would lead to cutaneous larva migration to the site of infection and inflammation [34]. Meanwhile, their clinical manifestations include eosinophilic enteritis, abdominal pain, diarrhea, and less frequent symptoms such as localized myositis and erythema multiforme, and ophthalmological complications may also occur [34,35]. *A. ceylanicum* is the only hookworm species that causes a patent infection, which is the second most common human ancylostomiasis in Asian countries [36,37]. *A. caninum*, which is the canine hookworm, remains the leading cause of human eosinophilic enteritis [36,38,39].

**Table-2:** The prevalence of identified worms infection in free roam dogs and its distribution in Bali Province.

Regency	Total sample	Prevalence					

		Ancylostomiasis	%	*Ascariasis*	%	*Trichuriasis*	%
Bangli	192	95	49.48	8	4.17	0	0.00
Badung	139	64	46.04	7	5.04	3	2.16
Denpasar	168	74	44.05	13	7.74	1	0.60
Tabanan	290	125	43.10	11	3.79	2	0.69
Gianyar	199	84	42.21	10	5.03	1	0.50
Negara	136	52	38.24	9	6.62	0	0.00
Karangasem	175	57	32.57	16	9.14	1	0.57
Klungkung	163	33	20.25	13	7.98	4	2.45
Buleleng	149	25	16.78	10	6.71	2	1.34
Total	1.611	609	37.80	97	6.02	14	0.87

**Table-3:** The intensity of *Ancylostoma* spp. infection in free-roam dogs and its distribution in Bali Province.

Regency	Total sample	Positive sample	Light	%	Moderate	%	Heavy	%
Denpasar	168	74	19	25.68	24	32.43	31	41.89
Badung	139	64	25	39.06	22	34.38	17	26.56
Gianyar	199	84	26	30.95	24	28.57	34	40.48
Tabanan	290	125	50	40.00	41	32.80	34	27.20
Bangli	192	24	43	66.67	26	25.00	26	8.33
Klungkung	163	57	16	31.58	6	38.60	2	29.82
Karangasem	175	52	18	48.08	22	36.54	17	15.38
Buleleng	149	25	11	44.00	13	52.00	1	4.00
Negara	136	95	25	45.26	19	27.37	8	27.37
Total	1.611	600	233	38.83	197	32.83	170	28.33

The prevalence of hookworm infection in free-roaming dogs in Bali (Tables-2 and 3) was higher than the prevalence rates of 33.03% in the Ilam Province of Iran [27], 20.23% in Guangdong, China [40], and 25% in Chittagong Metropolitan, Bangladesh [25]. However, it was lower than the prevalence rates of 48% in Malaysia (comprising 71.4% and 48% in rural and urban free-roaming dogs, respectively) [41], 40.5% in Central Italy [28], and 36.6% in the Ilam Province of Iran [27]. *Ancylostoma* spp. was not recorded in dogs in Northwest Mexico [29]. Furthermore, it was observed that 10%–12% of hookworm infection intensity was moderate (EPG 3000–10,000) and heavy (EPG > 10,000) (Table-3). Adult worms feed on the blood from the mucosa of the small intestine by opening vessels with their toothed buccal capsule. Therefore, it is the parasitic burden that causes the lesions in the host tissues. Ancylostomiasis causes secondary acute or chronic hemorrhagic anemia, and the amount of blood removed is directly proportional to the weight of the adult parasite [42]. In a mild infection, an iron deficiency will develop microcytic hypochromic anemia [33].

Dogs play a key role in the transmission of *Toxocara* spp., which are directly transmitted from pets to the human environment without the involvement of vectors or intermediate hosts [43]. Meanwhile, infection occurs when humans accidentally ingest the infected eggs, with children being the most vulnerable [10,44]. Two species of *Toxocara* that has been reported to infect dogs are *T. canis* and *Toxascaris leonine* [30]. The prevalence of *Ascaris* spp. in Bali province (Tables-2 and 4) are lower than the prevalence rates of 23.33% reported in Chittagong Metropolitan, Bangladesh [25], 21% in Ethiopia [9], and 20.6% in Central Italy [28]. However, it was higher than the prevalence of 5% reported in Mexicali County [29].

**Table-4:** The intensity of *Ascaris* spp. infection in free-roam dogs and its distribution in Bali Province.

Regency	Total sample	Positive sample	Light	%	Moderate	%	Heavy	%
Denpasar	168	13	7	53.85	5	38.46	1	7.69
Badung	139	7	2	28.57	5	71.43	0	0.00
Gianyar	199	10	7	70.00	1	10.00	2	20.00
Tabanan	290	11	5	45.45	5	45.45	1	9.09
Bangli	192	6	7	66.67	1	0.00	0	33.33
Klungkung	163	16	4	50.00	0	18.75	2	31.25
Karangasem	175	9	8	44.44	3	33.33	5	22.22
Buleleng	149	10	6	60.00	2	20.00	2	20.00
Negara	136	8	4	87.50	3	12.50	2	0.00
Total	1,611	90	50	55.56	25	27.78	15	16.67

*Trichuris* spp. are known to cause infection in humans. Moreover, few clinical cases that were triggered by *Trichuris vulpis* have been reported in Thailand, USA, and Mexico [45]. The prevalence of *Trichuris* spp. in free-roaming dogs in Bali (Tables-2 and 5) was different from the reports in Bangladesh and Central Italy at 45% [25] and 17.6% [28], respectively. Compared with *T. vulpis*, *Trichuris serrate*, and *Tapeinosperma campanula* are the two species of *Trichuris* that can infect cats. However, previous studies have reported a low prevalence of whipworm in cats [46].

**Table-5:** The intensity of *Trichuris* spp. infection in free-roam dogs and its distribution in Bali Province.

Regency	Total sample	Positive sample	Light	%	Moderate	%	Heavy	%
Denpasar	168	1	1	100.00	0	0.00	0	0.00
Badung	139	3	2	66.67	1	33.33	0	0.00
Gianyar	199	1	1	100.00	0	0.00	0	0.00
Tabanan	290	2	1	50.00	1	50.00	0	0.00
Bangli	192	3	0	66.67	0	0.00	0	33.33
Klungkung	163	1	2	100.00	0	0.00	1	0.00
Karangasem	175	0	1	0.00	0	0.00	0	0.00
Buleleng	149	2	0	0.00	1	50.00	1	50.00
Negara	136	0	0	0.00	0	0.00	0	0.00
Total	1.611	13	8	61.54	3	23.08	2	15.38

This study also revealed the presence of multiple infections among the worms. The combination of *Ancylostoma* spp. and *Toxocara* spp. was the most common combined infection among all three STH cases (Table-6). However, hookworm and roundworm are the most important parasites affecting dogs worldwide in terms of dispersal as well as risk for animal and human health [10]. In general, this polyparasitism affects not only the body condition, survival, or reproduction but also the metabolism, genetics, or immune investment of the host [47]. Interactions among parasites strongly influence their dynamics and therefore play a major role in structuring populations [48].

**Table-6:** The multiple infections of soil-transmitted helminths in free-roam dogs and its distribution in Bali Province.

Regency	Total sample	A+B	%	A+C	%	B+C	%	A+B + C	%
Bangli	192	10	5.95	1	0.60	0	0.00	0	0.00
Badung	139	6	4.32	3	2.16	0	0.00	0	0.00
Karangasem	175	11	6.29	0	0.00	0	0.00	0	0.00
Denpasar	168	10	5.03	1	0.50	0	0.00	0	0.00
Klungkung	163	6	4.03	0	0.00	1	0.67	0	0.00
Negara	136	3	1.84	2	1.23	0	0.00	1	0.61
Gianyar	199	6	3.13	0	0.00	0	0.00	0	0.00
Buleleng	149	4	2.94	0	0.00	0	0.00	0	0.00
Tabanan	290	6	2.07	1	0.34	0	0.00	1	0.34
Total	1611	62	3.85	8	0.50	1	0.06	2	0.12

A=*Ancylostoma* spp., B=*Ascaris* spp., C=*Trichuris* spp.

Moreover, the incidence of infections with different parasite species is generally increasing at the geographical scale [49]. However, there is still a scarcity of investigations in disease and evolutionary ecology on the impacts of multiple infections on individual hosts or higher parasite species richness on populations [47,49].

The Bali area consists of both highland and lowland, although primarily lowland, and highland is found only in Tabanan, Bangli, and Singaraja regencies. Our analysis also showed that the differences in altitude had no effect on the prevalence of STH infection in free-roaming dogs in Bali (Table-7). Therefore, this finding differs from the case of helminthiasis in rodents that were strongly influenced by landscape characteristics [50].

**Table-7:** The prevalence of soil-transmitted helminths infection according to the landscape.

Parasite	Landscape	Results		Total	p value

		Negative	Positive		
STH	Highland	209	124	333	0.139
	Lowland	758	520	1278	
	Total	967	644	1611	
*Ancylostoma* spp.	Highland	211	122	333	0.335
	Lowland	791	487	1278	
	Total	1002	609	1611	
*Ascaris* spp.	Highland	318	15	333	0.118
	Lowland	1196	82	1278	
	Total	1514	97	1611	
*Trichuris* spp.	Highland	332	1	333	0.180
	Lowland	1265	13	1278	
	Total	1597	14	1611	

## Conclusion

The prevalence of STH infection in free-roaming dogs in Bali was high (38.36%). The STHs causing infection in dogs in Bali were *Ancylostoma* spp. (37.8%), *Ascaris* spp. (6.02%), and *Trichuris* spp. (0.87%). Moreover, polyparasitism occurs in multiple infections caused by *Ancylostoma* spp. and *Ascaris* spp. In addition, the difference in altitude does not affect the prevalence of STH infection. Therefore, it is reasonable to conclude that the high prevalence of STH infection in free-roaming dogs in Bali is consistent with the life cycle of the parasite in the investigated area.

## Authors’ Contributions

KKA: Designed the study, performed the laboratory work, analyzed the data, and wrote the ­manuscript. MSA: Designed the study, collected the samples, performed laboratory work, analyzed the data, and wrote the manuscript. NAANS: Collected the samples, performed laboratory work, analyzed the data, and wrote the manuscript. WARW: Collected the samples, performed the laboratory work, analyzed the data, and wrote the manuscript. JKA: Collected the samples and performed the laboratory work. WNFG: Analyzed the data, and wrote the manuscript. IBMO: Performed the laboratory work, and analyzed the data. MS: Performed the laboratory work, and data analysis. NKB: Analyzed the data, and wrote the manuscript. All authors read and approved the final manuscript.
